# Synthesis and Characterization of a Novel Composite Edible Film Based on Hydroxypropyl Methyl Cellulose Grafted with Gelatin

**DOI:** 10.3390/gels9040332

**Published:** 2023-04-14

**Authors:** Yajuan Wang, Shuting Jiang, Yue Chen, Dan Qiu, Yunxuan Weng

**Affiliations:** 1 School of Material and Chemical, Ningbo University of Technology, Ningbo 315211, China; wangyajuan@nbut.edu.cn (Y.W.);; 2 Zhejiang Institute of Tianjin University, Ningbo 315201, China; 3 Beijing Key Laboratory of Quality Evaluation Technology for Hygiene and Safety of Plastics, Beijing Technology and Business University, Beijing 100048, China

**Keywords:** gelatin, hydroxypropyl methyl cellulose, structural properties, graft copolymer

## Abstract

A novel composite edible film was synthesized by grafting gelatin chain onto hydroxypropyl methyl cellulose (HPMC) in the presence of glycerol (used as a plasticizer) using a solution polymerization technique. The reaction was carried out in homogeneous aqueous medium. Thermal properties, chemical structure, crystallinity, surface morphology, and mechanical and hydrophilic performance changes of HPMC caused by the addition of gelatin were investigated by differential scanning calorimetry, thermogravimetric, Fourier transform infrared spectroscopy, scanning electron microscopy, X-ray diffraction, universal testing machine and water contact angle. The results shows that HPMC and gelatin are miscible and the hydrophobic property of the blending film can be enhanced with the introduction of the gelatin. Moreover, the HPMC/gelatin blend films are flexible, and exhibit excellent compatibility, good mechanical properties and also thermal stability, and could be promising candidates for food packaging materials.

## 1. Introduction

In the modern food industry, packaging materials are the “protective umbrella” for most food, keeping it fresh and safe during the manufacture process. This allows the food to be transported for long distances, and provides convenience to consumers. Food packaging materials account for up to 70% of the entire packaging industry field. Traditional food packaging materials are mostly synthetic polymers, commonly known as plastic bags, mainly produced from petroleum cracking products. However, this kind of material is difficult to degrade or may even be non-degradable. In China, the total output of plastic products reached 75.15 million tons in 2017. The annual disposable plastic packaging is about 80 million tons, which induces enormous pressure on the environment and resources. On the other hand, as a non-renewable energy source, the decline in petroleum reserves is increasingly apparent [1]. At the same time, hazardous substances with low molecular weight in synthetic polymer materials may migrate onto the surface of food, which leads to their insecurity as food packaging materials. Therefore, the preparation of films and coating materials from food-grade biopolymers is a common solution in the food packaging field. Recently, the literature has also shown that it has become a major trend to use biodegradable and edible raw materials to replace to traditional synthetic polymer materials in the food industry. The frequently used degradable materials contain polyester-based biomaterials, polyglycolic acid, polycaprolactone and plant-protein-based biomaterials [2]. 

Among these biodegradable raw materials, cellulose-composite packaging materials have occupied an important position in the field of food packaging due to their extensive sources, high biocompatibility and non-toxic environmental friendliness. In view of the presence of abundant free hydroxyl groups, cellulose can participate in various reactions to obtain its derivatives with different properties. With the development of nanotechnology, nano-cellulose composite films are emerging [3]. Hydroxypropyl methyl cellulose (HPMC) is a common cellulose derivative with some methyl and hydroxypropyl groups, which promote its water solubility. Therefore, it has been used in the food industry as an emulsifier, preservative, thickener, stabilizer and film-forming material [4]. As a kind of edible plant-based raw material derivative, HPMC can form a transparent, oil-resistant, odorless, tasteless and water-soluble film. The film exhibits good mechanical properties, acts as an effective lipid oxygen barrier and has moderate resistance to water vapor transfer [5,6]. However, due to its high price and poor water vapor barrier properties, composite HPMC films formed with other biopolymers ( such as polyacrylamide or starch and its derivatives), have been utilized in many industrial circles for use in controlled/sustained drug delivery, medical capsules and packaging materials [7,8,9].

Gelatin (Gel) is a partial hydrolysate product of collagen comprising Gly-Pro-Hyp sequences which is normally found in most connective tissue. Gelatin, which derived from living organisms, is widely used in many fields, and can be used in the chemical, pharmacy and food industries due to its high biocompatibility, weak antigenicity, bioactivity and good biodegradability [10]. Because of their excellent elasticity and edible security, gelatin-based films still play an important role in controlled-release film, edible sausage casing and soft capsule coating [11,12,13]. Nevertheless, gelatin film has some disadvantages in food packaging properties; for example, it has low thermostability and is hard and brittle, which limits its applications.

In summary, single-material-based packaging films would be inefficient in maintaining the strength and rigidity which are normally required. Earlier studies have concluded that blended film materials based on two or three components display excellent properties. For example, after blending, HPMC can hinder the recrystallization of starch-based material [6,14,15,16,17]. In addition, a blended film based on collagen and HPMC has also been studied; this research showed that the thermal and morphological properties and mechanical strength of the composite film (collagen/HPMC 1/1) are better than those of pure collagen film.

However, so far, there is no research on HPMC and Gel composites, and there is no systematic research about the impact of different ratios between HPMC and gelatin on the structure and properties of HPMC/Gel composite films that can be applied to food packaging materials. The objective of this work was to prepare a novel composite edible film based on HPMC and Gel. The effect of % HPMC and % Gel on the thermal stability, mechanical properties, reaction mechanism, morphology and hydrophilic performance of the composite material was also studied.

## 2. Results and Discussion

### 2.1. DSC

**Figure 1:**
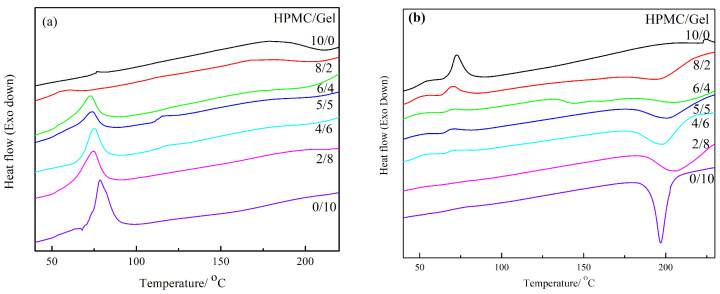
The DSC curves of all samples: (**a**) natural dry; (**b**) freeze dry.

Figure 1 shows DSC profiles of blended films with two different dry methods. For the most natural semi-crystalline polymers containing both crystalline and amorphous structures, the glass transition temperature Tg and melting temperature Tm can be demonstrated in the DSC thermograms. However, it is difficult to distinguish Tg from the endothermic peak, because the endothermic relaxation is representative for polymeric materials in the glassy state that undergoes natural ageing [18]. Melting is an endothermic process and crystallization is exothermal; the Tm and exothermic peak exhibited at DSC thermograms of different blended films were related to the degree of crystallinity of each sample. In early literature, it has been reported that the blending of HPMC can decrease the melting point of poly(ethylene oxide) (PEO) because of the damage to the crystal structure of PEO through the reaction between the other polymer and PEO [15,19,20]. Similar to with PEO, grafting polymerization between HPMC and Gel also can reduce the denaturation temperature and endothermic enthalpy of pure Gel. According to one report, the dry temperature can affect the melting temperature of gelatin [21]. Furthermore, the Tm and melting enthalpy is attributed to the helix-coil transition of gelatin [22]. Therefore, the endothermic enthalpy will increase with the increase in the Gel ratio in the blended film in the natural drying conditions (20 °C, Figure 1a).

Compared with natural drying, the temperature during freeze-drying is lower and the structural damage to the materials is smaller. Comparing Figure 1a,b, it can be seen that the freeze-dried samples have an obvious exothermal peak at a high temperature except for the pure HPMC film, which could be attributed to the cold crystallization of the freeze-dried blended films. With pure protein, different drying methods can affect its thermal stability [23], and the freeze-dried protein exhibited a higher endothermic denaturation peak than the natural-dried sample. Similarly, the freeze-dried HPMC/Gel shows an obvious exothermal peak at a high temperature, and the natural-dried sample exhibits an endothermic peak at a low temperature in our work, which indicates the composite material is more resistant to heat after freeze-drying with the increase in gelatin. Therefore, the freeze drying method is more suitable for packaging films that are used with high-temperature food.

### 2.2. TGA

**Figure 2:**
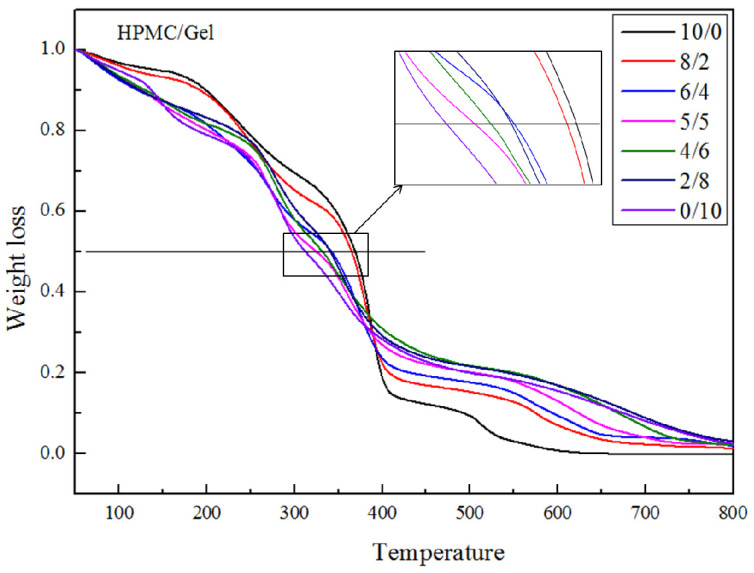
TGA thermogram of blending films.

TGA determines the mass change as a function of temperature and is always used to test the thermolysis of a sample as well as the residual ash. The TGA curves which characterized the thermal decomposition of all films in N_2_ are presented in Figure 2.

As can be seen from the TGA curves in Figure 2, pure HPMC presents two main stages in its degradation pattern. However, before its thermal degradation, a small weight loss at 50 to 140 °C can be observed for the HPMC sample due to the vaporization of moisture. In addition, the first main stage of thermal destruction began at about 160 °C (T_onset 1_), in which the weight loss percentage was 12%, and the rate of maximum weight loss occurred at 233 °C. At this temperature (160 °C), the plasticizer (glycerol) began to volatilize. The following stage occurred between 350 and 480 °C, and this thermal weight loss was mainly put down to the breakage of the cellulose ether bonds, which included the simultaneous processes of chain-scission and demethoxylation. Compared with the literature, the TGA curve of pure HMPC in our experiment has different thermal degradation behavior, mainly because of the different degree of substitution of HPMC and the different preparation methods of HPMC blended film [24]. During this experiment, part of the hydroxypropyl and methyl groups of HPMC may have broken away from main chain in the process of heating.

For the Gel film, the thermal degradation is mainly divided into four steps. For the pure Gel film, the first stage of thermal decomposition occurred between 50 °C and 140 °C. The weight loss ratio was approximately 12%, and this loss was caused by water loss in the gelatin film structure. It can be seen that the fastest pyrolysis temperature was 145 °C for the second step and the Gel film lost about 11% of its mass. Similar to with pure HPMC, the weight loss in this stage is deemed to be the thermal decomposition of the glycerol. The following two steps have no clear boundary; and the maximal degradation temperatures are 283 °C and 359 °C, respectively, and the weight-loss percentage is about 60%. The weight loss is caused by the thermal decomposition of the gelatin polymer chain [25,26].

Compared with the Gel film, the HPMC has a higher thermal stability. Therefore, the thermal degradation of the blended film is in between that of pure HPMC and Gel films. Generally, for polymers, the decomposition temperature when the weight loss is 50% is called the semi-life temperature. A higher semi-life temperature indicates that the polymer has a better thermal stability. According to Figure 2, the semi-life temperature of the blended film will increase with the increase in HMPC. These results showed that the addition of HPMC can increase the thermal stability of Gel film.

### 2.3. XRD Analysis

**Figure 3:**
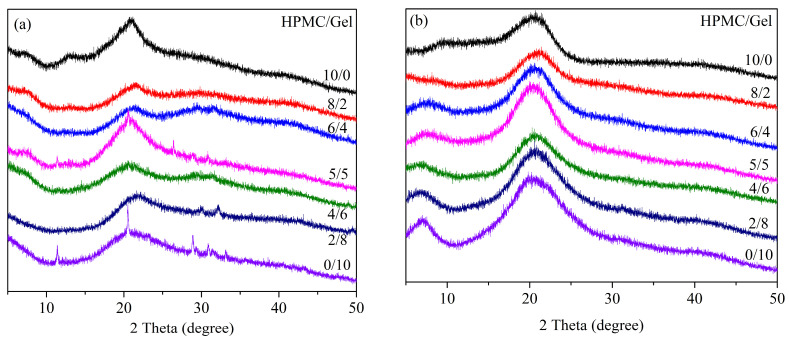
XRD patterns of HPMC and Gel blended film: (**a**) natural dry; (**b**) freeze dry.

The crystal texture of pure and blended HPMC films was also investigated by X-ray diffraction (Figure 3). For the pure HPMC film sample, it exhibited a largely amorphous with a broad undefined peak at about 20°, and two weak peaks at around 7.8° and 14.5°. According to the previous research, there are two peaks at around 7.8° and 20° (2θ) for pure HPMC film [27]. In our diffraction pattern, a new peak at 14.5° for the HPMC film sample should be attributed to the matrix formed between HPMC and the plasticizer (glycerol), which is also in good agreement with the results from literature [28]. Research has shown that collagen exhibited an obvious peak at around 7.5° (2θ), corresponding to the diameter of the triple helix structure, and a diffuse broad peak at about 20° (2θ), corresponding to the interval between amino acid residues along the helix of collagen [29,30]. As the hydrolysate of collagen, the pure Gel film exhibited a sharp peak at 11.4° (2θ) and 20.5° (2θ) for the naturally dried sample, and 7.0° (2θ) and 20.2° (2θ) for the freeze-dried sample, and three unresolved peaks at 28~35° (2θ) for the naturally dried Gel film. Therefore, the crystal form of polymer materials can be maintained by the freeze-drying method. As the ratio of Gel decreased in the blended film, the typical peak at 20° (2θ) became weakened, and the peak at around 12° (2θ) gradually faded for the naturally dried film. For the freeze-dried samples, the characteristic diffraction peak of Gel at 7.0° is weakened, and even disappeared for the sample HPMC/Gel 8/2. It is worth noting that regardless of the type of drying method, the crystallinity degree was found to be best in the HPMC/Gel 5/5 sample.

### 2.4. FT-IR of the Films

**Figure 4:**
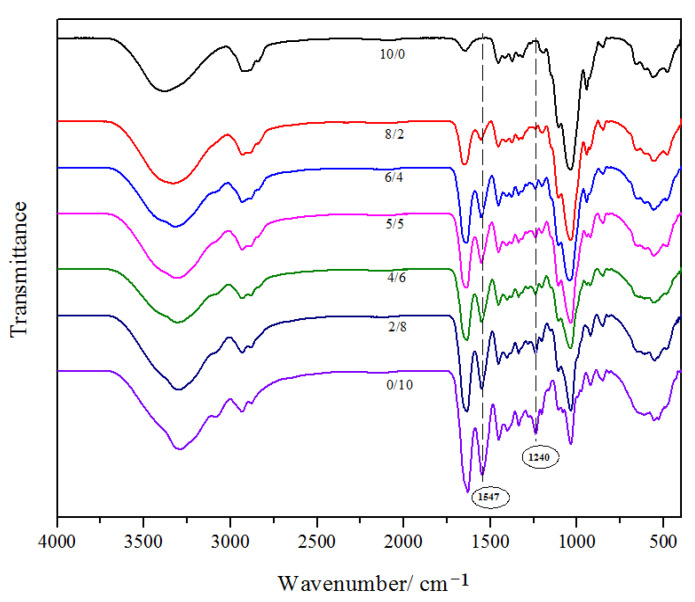
FTIR spectra of HPMC/Gel composite films.

Generally, FTIR test is a method that is sensitive to the structure and local molecular environment of polymers. In addition, the FT-IR spectra of all composite film with different HPMC and gelatin ratios exhibited similar characteristic peaks with different amplitudes, depending on the percentage of gelatin (Figure 4). The band situated near 3280 cm^−1^ was discovered in all specimens, corresponding to the stretching vibration of the hydroxyl groups (-O-H) existing in the molecule of both HPMC and gelatin. It is generally known that the characteristic absorption peaks of hydroxyl groups will shift to a lower frequency because of stretching vibration, especially when intermolecular and intramolecular hydrogen bonds linkages are formed [31]. As seen in Figure 4, the stretching vibration of the hydroxyl groups (-O-H) in the composite film moved from 3372 cm^−1^ to 3280 cm^−1^ due to the addition of gelatin, indicating the reaction between HPMC and gelatin molecules. For the pure HPMC film, the characteristic absorption bands were at 3460 cm^−1^ and 1040 cm^−1^ resulting from the stretching vibration of the hydroxyl group (O-H) and ether bond (C-O) groups, respectively [8]. Compared with pure HPMC membrane, the new peaks situated around 1547 cm^−1^ and 1240 cm^−1^ can be detected for the blended film, which was the characteristic peak of the amide II band (corresponding to the bending vibration of N-H) and amide III band (corresponding to the stretching vibration of C-N). It can be attributed to the peptide bond of the gelatin. Interestingly, the two-peak intensity is enhanced with the increase in the gelatin ratio in the composite film, which means the homogeneous recombination between HPMC and gelatin. Besides this, the peaks observed at around 1640 cm^−1^ indicate the C-O of the six carbon cyclic of HPMC, and phenylalanine or tyrosine of gelatin [32]. As the gelatin content increases, the intensity of the peaks near 1040 cm^−1^ weakens, and the intensity at around 1645 cm^−1^ increases obviously, which implies that HPMC and gelatin can be linked through the intermolecular hydrogen bonds in the composite film. Generally, if the compatibility between components in the composite material is poor, each polymer component shows its specific peak positions in the blended films. Conversely, there will be shifts in the wavelength due to the chemical interactions between different constituents if the polymers are miscible. In this research, there are significant differences between the spectra of single components and HPMC/Gel films, which is evidence of the miscibility of HPMC and Gel blends.

### 2.5. Mechanical Properties

The tensile strength and elongation at break of packing materials have a significant impact on their practical applications. The research of Fan et al. showed that the chemical reaction among different polymers’ components have an obvious influence on the mechanical properties of the composite polymer [33]. The effect of increases in the proportion of the Gel on the ultimate tensile strength and elongation at break of the HPMC/Gel composite films is shown in Table 1. From the data in Table 1, it can be seen that the increase in the Gel content significantly increased the ultimate tensile strength and decreased the elongation at break of the blended films, except for sample 5 whose tensile strength was 26.76 MPa and Gel content was 60%.

By reducing intermolecular forces, increasing the mobility of polymer chains and improving their flexibility, the addition of plasticizer can effectively reduce the inherent brittleness of composite films [5]. Increased crystallinity in the blended films contributes to the increased tensile strength. Compared with the commercial plastic packaging film HDPE (whose tensile strength is 25 ± 2 MPa), the tensile strength of HPMC/Gel composite film is better [34]. Overall, mechanical properties depend on the microstructural network, constituents, matrix filler interactions, preparation conditions, plasticizer and existing intermolecular force [35]. The changes in the mechanical properties can be attributed to the interaction of characteristics among the polymer components and rearrangement of the polymer network.

### 2.6. SEM

**Figure 5:**
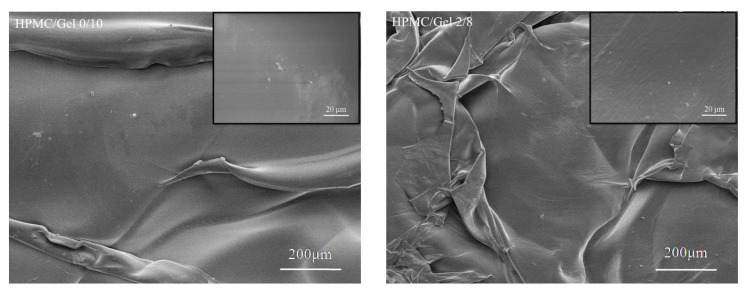

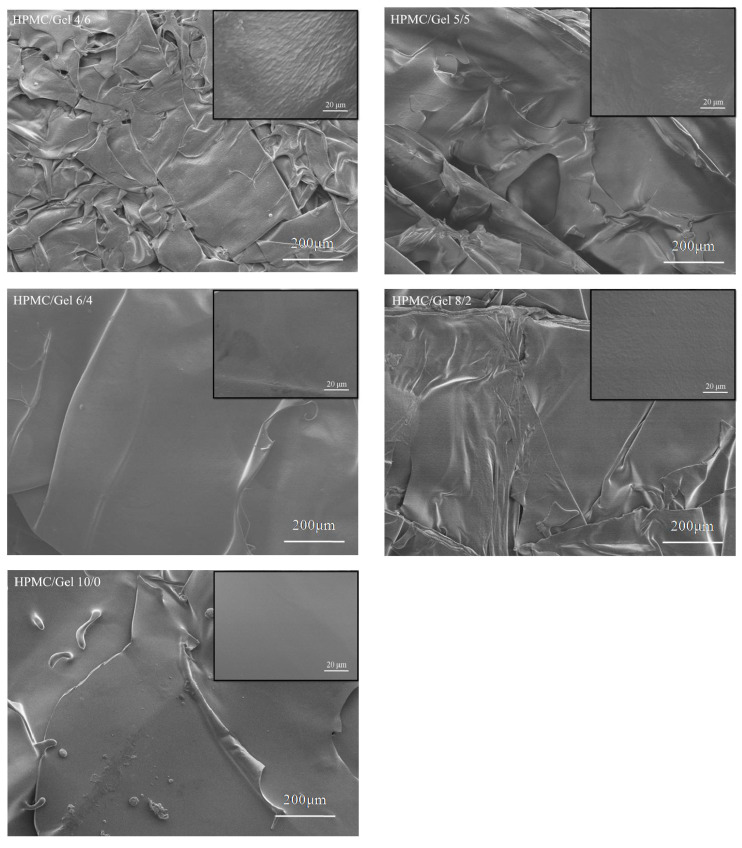
SEM images of freeze-dried blending film.

The microstructures of the longitudinal section of freeze-dried films detected by scanning electron microscope are shown in Figure 5. The longitudinal section of the pure Gel film showed an excellent homogeneous and compact structure compared with the blended and pure HPMC films, exhibiting a good property of film-forming [36]. In addition, the surfaces of all the HPMC and Gel blended films are uniform, meaning that the blended films did not show any microscopic phase separation. Two components in the composite film have abundant hydrophilic groups and could be linked by the chemical bonding between gelatin or HPMC and glycerol molecules. The results suggested that the HPMC and Gel have a good compatibility.

### 2.7. Water Contact Angle

The measurement of the water contact angle (WCA) provided evidence of the nature of the surface and the microstructure of the composite film, and the hydrophobicity and hydrophilicity of HPMC-based films (Table 2). The Gel film exhibited a hydrophobic nature characterized by a high WCA, i.e., 101.23 ± 0.13, whereas the HPMC film had a lower WCA value of 52.01 ± 0.63, which means the hydrophobicity of gelatin was much better than that of HPMC film. Similar conclusions have been summarized by Ding et al. [4]. The distribution and amount of hydrophilic groups on the surface of the polymer materials were the primary factors altering the WCA. As expected, the hydrophobicity of the HPMC/Gel composite membrane was visibly enhanced due to the introduction of hydrophobic groups from Gel, such as indolyl and phenyl groups [37]. Accordingly, the WCA of the blended films gradually increased with the increase in the proportion of the Gel constituent. This phenomenon, which reduces the hydrophilic property of the composite films, can be ascribed to the formation of Schiff’s base depleting the hydrophilic groups on the surface of the blending membrane [38]. A higher WCA represents a more effective resistance to water drop penetration [39]. Good hydrophilicity means that the water in food is more easily lost during storage, which then results in changes in taste and texture.

## 3. Conclusions

Gelatin–HPMC blended films were successfully produced by the graft copolymerization method in aqueous solution. The XRD and SEM images show that the Gel/HPMC blended film (5/5) has a good crystallinity degree, homogeneous size distribution and smooth structures. According to the DSC and TGA results, it can be seen that the addition of gelatin could significantly improve the thermal stability of HPMC. The chemical structure of HPMC slightly changed after blending with gelatin. Meanwhile, the hydrophobic property of HPMC material can be improved with the addition of gelatin, suggesting that HPMC/Gel blended films have the potential to substitute traditional paper-plastic package materials. In addition, the mechanical properties of the blended films were influenced by the gelatin content, and the Gel/HPMC blended film (5/5) had a good tensile strength. These results show that the HPMC/Gel blended film material could become a novel material with potential applications, especially in the food packaging field, because of its excellent properties such as low cost, green initiative and being easy to apply.

## 4. Materials and Methods

### 4.1. Materials

A commercially available, food-grade HPMC (average Mn~86,000, 1.8~2.0 mol methoxy per mol cellulose, Fisher Scientific Chemicals Company, Pittsburgh, PA, USA) was used in this work. Gelatin (type A, from pigskin) and glycerol (analytically pure) were purchased from Sigma-Aldrich Chemicals Company (St. Louis, MO, USA). All other chemicals used were analytically pure and obtained from Fisher Scientific Co. (Pittsburgh, PA, USA), without further purification.

### 4.2. Preparation of the HPMC/Gel Composite Material

The HPMC and gelatin were mixed in a three-necked bottle, and the HPMC:Gel weight ratios were 10:0, 8:2, 6:4, 5:5, 4:6, 2:8 and 0:10. The required amount of water was added into the bottle to ensure the mass fraction was 10%. The three-necked bottle was stirred for 12 h at 25 °C. The pH of the mixture was adjusted to 6.8, and glycerol (20%, based on polymer total weight percentage) was gradually added dropwise into the bottle with gentle stirring, and then the temperature was increased to 85 °C for 2 h in order to obtain a homogeneous gelatin solution. Following this, the mixed solution was cooled to 25 °C to dissolve the HPMC with stirring at 100 rpm for 1 h. After the reaction was completed, 15 g of the solution was poured onto a rounded dish with a diameter of 10 cm, and dried at room temperature to obtain the film. The blended films were prepared by pouring into a petri dish and freeze-drying at −50 °C for 48 h.

### 4.3. X-ray Diffraction Measurements

In order to ascertain the crystalline nature of the prepared composite materials, the X-ray diffraction studies were carried out on a Bruker D8 advance X-ray diffraction meter. The samples were scanned in the 2θ range from 5° to 60°. The scanning speed and step size were 0.3°/min and 0.01°, respectively. The operating target voltage was 40 kV, tube current was 100 mA and the radiation used was with Ni-filtered CuK α radiation of wavelength λ = 1.5406 Å. The intensity versus scans was obtained for different samples.

### 4.4. Fourier Transform Infrared Spectroscopy

FTIR spectra of all films were collected with a Nicole 6700 FT-IR Spectrometer (Thermo Fisher Scientific Co., Waltham, MA, USA). A total of 64 scans were run for every sample in the wave number range from 400 to 4000 cm^−1^ with resolution 4 cm^−1^ at 25 °C. The FTIR spectrum was taken in a transmittance mode.

### 4.5. Thermogravimetric Analysis

The thermal behavior of the scaffold materials was studied using a Perkin-Elmer thermal gravimetric analyzer (Pyris 1, Norwalk, CT, USA). The weight of every sample was about 5 mg, and all measurements were taken under a dry N_2_ atmosphere at 60 mL/min. All films were heated up to 700 °C from 50 °C with a heating rate of 20 °C/min.

### 4.6. Differential Scanning Calorimetry

The thermal properties were performed using a German NETZSCH 204DSC under nitrogen purge. Approximate 4 mg samples were accurately weighted and sealed hermetically in a high-volume stainless steel pan. Samples were heated from 30 °C to 250 °C with a heating rate of 10 °C/min. The area of the peak was determined in the DSC thermograms. Every sample measurement was performed in triplicate.

### 4.7. Mechanical Properties

The tensile strength (TS) and elongation at break of the all strip-shaped samples were determined to evaluate the mechanical properties using a CMT4204 Tensile Machine (MTS Industrial Systems Co. LTD, Eden Prairie, MN, USA). First of all, the samples were cut into rectangles of 5 mm wide and 100 mm long using a specific mold. Then, the strip-shaped samples were equilibrated in a 25 °C dryer with 65% relative humidity for 24 h. Subsequently, each specimen of length 50 mm was gripped, and then the samples were uniaxially stretched at a constant speed of 2 inch/min in the vertical direction. The tensile strength and elongation at break were recorded. The measurement was repeated three times for every sample to calculate the average value.

### 4.8. Scanning Electron Microscopy

The morphology of the freeze-dried HPMC/Gel scaffolds was observed by scanning electron microscopy (SEM, Hitachi S4800, Hitachi, Ltd., Tokyo, Japan). In brief, all the samples were fractured in liquid N_2_, sputter-coated with aurum at 8 mA for 30 s and imaged at an accelerating voltage of 10 kV.

### 4.9. Water Contact Angle Tests

The wettability characteristic of a solid surface is usually evaluated by the sessile drop method with a contact angle meter (KSV CAM 100 Optical Contact Angle Meter, Stuttgart, Germany). Firstly, each film specimen (30 mm × 20 mm) was fixed on a glass slide. Then, a distilled water droplet of 7 μL was carefully deposited onto the surface of a sample. Finally, according to the droplet boundary tangent and droplet baseline, the left and right contact angles were calculated using Krüss drop shape analysis software (Hamburg, Germany). The average WAC was recorded by measuring each sample three times. In order to ensure the accuracy, all tests were conducted at room temperature (25 ± 1 °C).

### 4.10. Statistic Analysis

All statistical analyses were conducted using SAS software (v.9.4, SAS Institute, Cary, NC, USA). The data from experiments were analyzed through analysis of variance (ANOVA).

## Figures and Tables

**Table 1:** Mechanical Properties of HPMC and its blended films.

Sample No.	HPMC/Gel	Tensile Strength (MPa)	Elongation at Break (%)
1	10/0	26.08 ± 1.51	23.2 ± 2.1
2	8/2	28.05 ± 0.99	21.7 ± 3.2
3	6/4	29.58 ± 0.36	18.9 ± 1.4
4	5/5	32.93 ± 1.37	16.9 ± 2.3
5	4/6	26.76 ± 0.74	16.2 ± 2.2
6	2/8	33.42 ± 1.29	16.1 ± 2.1
7	0/10	42.69 ± 1.36	9.4 ± 1.2

**Table 2:** Water contact angle of HPMC-based composite films.

HPMC/Gel	WCA (°) (Mean ± SD)
10/0	52.01 ± 0.63
8/2	61.22 ± 0.99
6/4	67.05 ± 0.14
5/5	85.13 ± 0.56
4/6	86.41 ± 0.90
2/8	92.46 ± 0.20
0/10	101.23 ± 0.13

## Data Availability

The data that support the findings of this study are available within the article.
